# Supporting front crawl swimming in paraplegics using electrical stimulation: a feasibility study

**DOI:** 10.1186/s12984-020-00682-6

**Published:** 2020-04-16

**Authors:** Constantin Wiesener, Lotta Spieker, Jens Axelgaard, Rachel Horton, Andreas Niedeggen, Nikolaus Wenger, Thomas Seel, Thomas Schauer

**Affiliations:** 1 Technische Universität Berlin, Control Systems Group, Einsteinufer 17, Berlin, Germany; 2 Axelgaard Manufacturing Co., Ltd., Fallbrook, USA; 3 Treatment Centre for Spinal Cord Injuries, ukb Unfallkrankenhaus Berlin, Warener Str. 7, Berlin, Germany; 4 Department of Neurology, Charité - Universitätsmedizin Berlin, Charitéplatz 1, Berlin, Germany

**Keywords:** Swimming, Exercise, Functional electrical stimulation (FES), Transcutaneous spinal cord stimulation (tSCS), Spinal cord injury (SCI)

## Abstract

**Background:**

Participation in physical and therapeutic activities is usually severely restricted after a spinal cord injury (SCI). Reasons for this are the associated loss of voluntary motor function, inefficient temperature regulation of the affected extremities, and early muscle fatigue. Hydrotherapy or swim training offer an inherent weight relief, reduce spasticity and improve coordination, muscle strength and fitness.

**Methods:**

We present a new hybrid exercise modality that combines functional electrical stimulation (FES) of the knee extensors and transcutaneous spinal cord stimulation (tSCS) with paraplegic front crawl swimming. tSCS is used to stimulate the afferent fibers of the L2–S2 posterior roots for spasticity reduction. By activating the tSCS, the trunk musculature is recruited at a motor level. This shall improve trunk stability and straighten the upper body. Within this feasibility study, two complete SCI subjects (both ASIA scale A, lesion level Th5/6), who have been proficient front crawl swimmers, conducted a 10-week swim training with stimulation support. In an additional assessment swim session nine months after the training, the knee extension, hip extension, and trunk roll angles where measured using waterproof inertial measurement units (IMUs) and compared for different swimming conditions (no stimulation, tSCS, FES, FES plus tSCS).

**Results:**

For both subjects, a training effect over the 10-week swim training was observed in terms of measured lap times (16 m pool) for all swimming conditions. Swimming supported by FES reduced lap times by 15.4% and 8.7% on average for Subject A and Subject B, respectively. Adding tSCS support yielded even greater mean decreases of 19.3% and 20.9% for Subjects A and B, respectively. Additionally, both subjects individually reported that swimming with tSCS for 30–45 minutes eliminated spasticity in the lower extremities for up to 4 hours beyond the duration of the session. Comparing the median as well as the interquartile range of all different settings, the IMU-based motion analysis revealed that FES as well as FES+tSCS improve knee extension in both subjects, while hip extension was only increased in one subject. Trunk roll angles were similar for all swimming conditions. tSCS had no influence on the knee and hip joint angles. Both subjects reported that stimulation-assisted swimming is comfortable, enjoyable, and they would like to use such a device for recreational training and rehabilitation in the future.

**Conclusions:**

Stimulation-assisted swimming seems to be a promising new form of hybrid exercise for SCI people. It is safe to use with reusable silicone electrodes and can be performed independently by experienced paraplegic swimmers except for transfer to water. The study results indicate that swimming speed can be increased by the proposed methods and spasticity can be reduced by prolonged swim sessions with tSCS and FES. The combination of stimulation with hydrotherapy might be a promising therapy for neurologic rehabilitation in incomplete SCI, stroke or multiples sclerosis patients. Therefore, further studies shall incorporate other neurologic disorders and investigate the potential benefits of FES and tSCS therapy in the water for gait and balance.

## Background

A spinal cord injury (SCI) is typically associated with a paralysis of the lower extremities, which implies a major restriction of physical activity and health of the affected subjects. Depending on the level and severity of the injury, SCI entails a functional limitation of various sensory and motor functions below the level of the lesion. Furthermore, in the case of a traumatic SCI, the abrupt physical inactivity is in stark contrast to the condition prior to the injury, especially for young patients.

Due to the loss of voluntary motor function and inefficient temperature regulation of the affected extremities, autonomic dysfunction, and early muscular fatigue, the participation in physical and therapeutic activities following paraplegia is often limited. Specially adapted equipment and specifically trained therapists are needed. Despite all these obstacles, sportive and therapeutic activity after paraplegia can contribute to a reduction in secondary complications and to an increase in the emotional well-being of those affected [1, 2].

In the majority of cases, paraplegia results in complete or incomplete paralysis of the lower extremities. Therefore, effective and safe lower-extremity training is limited and training exercises of the upper extremities are recommended such as arm-crank ergometer training, wheelchair ergometer training or swimming. These exercises can improve physical fitness by up to 25% if regularly conducted [3]. Mobility in the water is often the only experience of unaided body movement (except for the transfer in and from the pool) within the environment that most paralyzed patients enjoy. In addition, there is a plurality of therapeutic effects described in the literature, including an increase of muscle strength, improved coordination, reduction of spasticity and a reduction of contractures [4].

Functional electrical stimulation (FES) is used successfully in FES cycling or rowing [5, 6]. The corresponding muscles for knee extension and flexion as well as hip extension are stimulated depending on the crank or joint angle during cycling or triggered by a pull switch while rowing [7]. By the combination of arm and leg training, a significantly higher training effect can be achieved. In addition, an improvement in perfusion and lower limb bone density has been observed in some studies [5]. To reduce mobility-limiting spasticity, transcutaneous spinal cord stimulation (tSCS) has been used in individuals with SCI [8, 9].

There are only a few studies on swimming in paraplegics in particular, although it is a Paralympic sport since 1960. In [10] the effect on the cardiorespiratory capacity of high frequency swim training on SCI patients was evaluated. After three years an increase of four times compared to baseline was observed, while the control group (conventional land based training) had no significant increase in the cardiorespiratory capacity.

Besides paraplegic swimming, aquatic therapy (e.g aqua jogging, aquarobics or underwater treadmill training) is used for incomplete SCI rehabilitation. There are currently three systematic reviews of the benefits of aquatic therapy to patients with SCI published in [11–13]. All three reviews state that the current research quality is low due to the lack of randomized controlled trials in the specific field of clinical rehabilitation. Despite the different measures across all studies, aquatic exercise programs were found to have a positive impact on physical function in all studies. The reason might be that the aquatic environment directly promotes and maximizes the participants’ residual motor function, leading them to feel more independent in the aquatic environment after an adaptation period. The same results can be formulated for cardiorespiratory fitness [14–16]. In several studies with only few patients, improvement of cardiorespiratory fitness could be observed. A further finding of some studies was that exercising while submerged in warm water lowers the heart rate and enhances thermo-regulatory responses, thereby prolonging the SCI patient’s ability to exercise and thus increasing their aerobic capacity [14, 17]. Finally, [18] reported a significant reduction of muscle spasticity with a reduced dosage of baclofen after hydrotherapy. Additionally, [11] found several other studies showing an increased range of motion for the lower extremities and reduced spasticity.

Electrical stimulation in water has been proposed a hundred years ago in [19], where it was used for massage or transferring pharmaceuticals by placing one electrode on the body of the subject outside the water and the counter electrode inside the water. Nowadays the same technique is used to generate muscle contractions or tactile feedback as presented, for example, in [20]. In [21], we presented the first FES system that restored functional movements in swimming paraplegics by stimulating with special waterproof electrodes.

The first question regarding FES support of paraplegic swimming is which leg movement and swimming style can and should be assisted by FES. There are several preferable swimming styles for paraplegics depending on the lesion height and swimming skills. For normal breaststroke in unimpaired swimmers, the so-called frog kick is used as leg technique. It includes knee flexors and extensors, thigh adductors and abductors, gluteus maximus, and the plantar flexors. In [22], we found out that – due to the high number of involved muscle groups – a complex movement like the frog kick is currently not realizable with FES. The easier so-called flutter kick can be used for backstroke and crawl. It involves mostly the hip and knee extensors and flexors. In [23, 24] the knee angle for healthy non-expert swimmers during front crawl was analyzed. After a short and strong extension phase, a plateau phase can be observed where the knee joint is fully extended. During this plateau phase, the contra-lateral knee is flexed to 40-50 degrees and then directly extended to the same plateau phase. In preliminary tests in [22], we showed that the stimulation of gluteus maximus muscle is difficult to realize since the electrodes could not be placed precisely by paraplegics themselves without assistance and the stimulation-induced hip angle change was quite low. Furthermore, the hip position of a paraplegic in backstroke swimming depends on the level of control over the hip. In preliminary tests, we found out that the more flexed the hip is, the less propulsion can be achieved by stimulating the knee flexors and extensors. Patients who are proficient in backstroke and front crawl will therefore profit most from stimulation support of the flutter kick during front crawl swimming. The synchronization of the flutter kick with arm movements might be beneficial to improve the propulsion as proposed in [22, 25].

In the present contribution, we present a pilot study with two proficient front crawl swimmers (both ASIA A SCI, T5/6) who performed a 10-week swim training with stimulation support. In addition to the functional electrical stimulation, we applied tSCS in order to reduce spasticity and to increase trunk stability [26, 27]. Inertial measurement unit (IMU)-based motion analysis is further introduced to study joint angles of the lower limbs as well as roll angles at the lower and upper trunk during swimming. In a post-training assessment, this method has been applied to unterstand performance differences that have been observed when using the different support modalities during the training sessions. The suitability of reusable silicon electrodes for FES and tSCS stimulation in water has been investigated as well during the post-training assessment.

## Methods

### Functional electrical stimulation support

Based on previous work, we decided to use FES-induced flutter kicks for proficient front crawl swimmers. Furthermore, floats are attached to the ankles that lead to knee flexion and an upward movement of the ankle in a non-stimulated leg. On the one hand, this results in a more streamlined posture in the water. On the other hand, it implies that the desired knee movement can be realized by alternating between FES-induced knee extension and passive knee flexion caused by the floats. Hence, only two stimulation channels are needed. The quadriceps muscles of both legs are alternately stimulated where the stimulation electrodes were placed at the proximal part of the rectus femoris and the motor point of the vastus medialis of each leg. The stimulation, which is applied with stimulation pulse frequency of 25 Hz, is switched on and off at a rate of 1 or 2 Hz which results in approximately one or two leg kicks per arm stroke depending on the arm stroke frequency. The amplitude and pulsewidth can be varied in the ranges 0–100 mA and 0–500 *μ*s, respectively. Both values are increased/decreased simultaneously to control the generated muscle contraction.

### Transcutaneous spinal cord stimulation

**Fig. 1:**
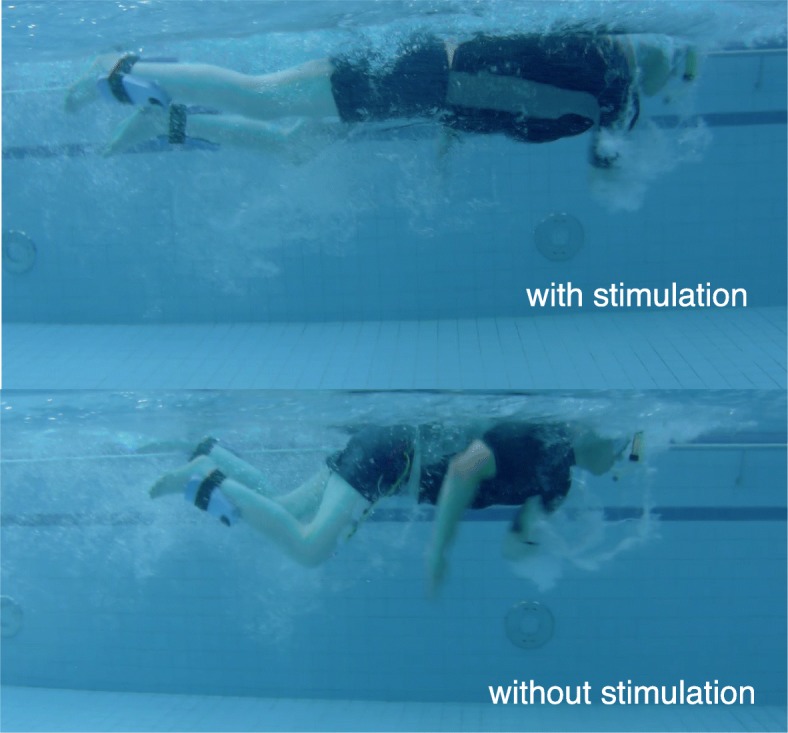
Paraplegic subject (Th5/6, ASIA scale A) with and without stimulation (FES+tSCS) using floats at the ankles and a snorkel. Comparing videos for swimming with and without stimulation support are available for both subjects of the study as supplementary files (See Additional files 1 & 2)


Transcutaneous spinal cord stimulation is used with the aim to reduce lower-limb spasticity during and after swimming. Therefore, we stimulate the afferent fibers of the L2–S2 posterior roots continuously at 50 Hz using biphasic pulses with 1 ms pulse width over the T11/12 region at the spinal cord according to [8]. The electrode position at the back and stimulation amplitude has been determined as outlined in [8]. By switching on the tSCS, the trunk musculature is activated at a motor level as a positive side effect. This improves trunk stability and straightens the upper body. As shown in Fig. 1, a streamlined swimming position can be achieved with FES and tSCS compared to no stimulation in a paraplegic subject.

Additional file 1: Subject A.


### Experimental setup

#### Stimulator

**Fig. 2:**
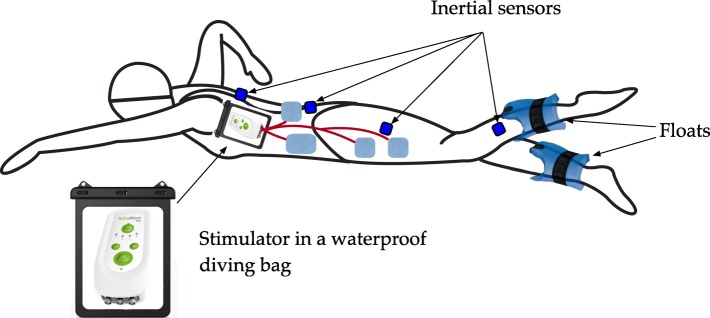
Stimulation-assisted swimming system including a waterproof stimulator, waterproof IMUs, floats at each shank, and waterproof electrodes

The stimulation system for swimming shown in Fig. 2 employs a CE-certified stimulator (RehaMove3, Hasomed GmbH, Germany) with customized firmware. A single current source is integrated into the device, and the output of the source is demultiplexed for up to 4 channels. The stimulator is placed inside a waterproof bag under the swimmer’s T-shirt. All stimulation cables are tunneled through the bag and drained with silicone to prevent water intrusion. The bag is attached with a strap on the swimmer’s back between the shoulder blades.

The stimulator can be controlled via the membrane keypad e.g. the stimulation program can be selected, started/stopped and the stimulation intensity can be adjusted. The stimulator is battery-powered, and the high-voltage source is galvanically isolated from the battery power. Hence, the current conduction is always constrained between the positive and the negative electrode of each stimulation channel.

#### Waterproof stimulation electrodes

Due to the fact that chlorinated water in swimming pools has a conductance of 2.5–3mS/cm, which results in resistance of 333–400 Ohm, a direct stimulation with non-waterproof electrodes would produce a parasitic short circuit between electrodes during stimulation. Therefore, the device-integrated electrode error detection might not detect a bad connection between the electrode and the skin. If both electrodes float in water, then the muscles would not be stimulated, because the current always takes the path of least resistance directly through the water and not the body. If only one electrode floats in water, then the current will still pass through the remaining firmly attached electrode and will still cause a muscle contraction beneath this electrode. The only potentially dangerous situation would occur when the conductive side of a detached and floating electrode would accidentally be firmly pressed against skin of the upper body, since then electrical currents might flow through sensitive organs, such as the heart. To minimize this risk and because of the limited electrode error detection, the electrodes need to be safely and firmly attached to the skin. Furthermore, the electrode side facing away from the body needs to be isolated against water. Possible measures are waterproof transparent film dressing, straps or swimming cloths.

Currently, there are no waterproof stimulation electrodes available on the market. Most transcutaneous electrodes consist of a conductive hydrogel adhesive which is connected via conductive film to a lead wire or metal snap stud and isolated with an insulative cover. If the hydrogel adhesive gets into contact with water it starts to absorb water while the thickness increases. Hence, the area with direct contact to the water increases. Furthermore, the adhesive function of the electrode is reduced. Approaches for underwater EMG measurement in [28, 29] used several layers of waterproof wound plaster with tunneled holes for the lead wires to waterproof standard adhesive EMG electrodes. The same procedure can be used for stimulation electrodes where standard electrodes are waterproofed with adhesive films, like Tegaderm^TM^ or OpSite^TM^.

**Fig. 3:**
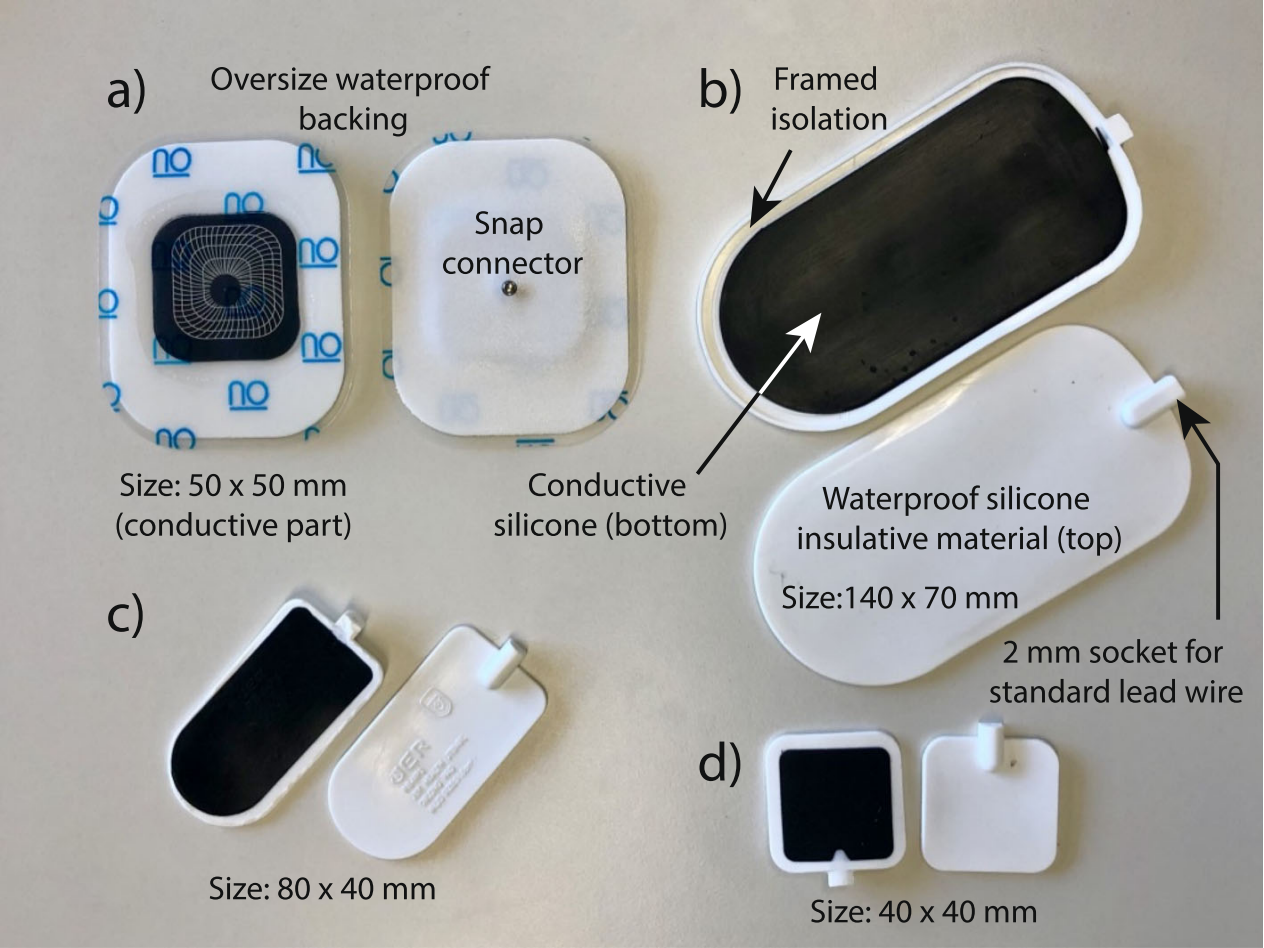
Electrodes used in water: **a** Axelgaard Ultrastim®snap electrode with oversize waterproof backing with an electrode area of 22.9 cm^2^ [30, 31] for tSCS (4 electrodes electrically connected for the abdomen and one over the spine) and FES (two electrodes for each quadriceps), **b** to **d** Safety silicone electrodes (VITAtronic Limited, Germany) consisting of an insulative and waterproof cover material and a conductive bottom material for tSCS (2 x (b) electrically connected for the abdomen and 1 x (d) for the back) and for FES (2 x (c) for each quadriceps)

For the training sessions of our pilot study, which is described in the next subsection, special electrodes developed by Axelgaard Manufacturing Co. Ltd have been used, as shown in Fig. 3a. A single electrode consists of a standard electrode with an oversized waterproof backing. The snap adapter is tunneled through this backing. The remaining task is then to connect the electrode lead (converter from the snap adapter to 2 mm socket) and seal it with a waterproof transparent film dressing (3M Tegaderm, 3M Co., USA). All cables and cable connections have to be waterproof as well. Otherwise, parasitic short circuits occur. Removable tight silicone tubes showed to be efficient in covering the connection between the electrode lead and the stimulation cable.

A drawback of adhesive electrodes with oversized waterproof backing is that after a single contact with water they cannot be reused. Hence, for each swimming session, a new set of electrodes is needed. To reduce costs and to save the environment, the suitability of reusable safety silicone electrodes shown in Fig. 3b to d has been investigated in a post-training assessment session. These electrodes are available in different sizes (VITAtronic Limited, Germany) and can be directly connected via a standard 2 mm electrode connector to the simulation cable. Due to the non-conducting upper side and the framed isolation on the conductive skin side, no parasitic short circuit can occur when firmly attaching the electrodes to the skin. The material is non-adhesive, which reduces skin irritation during the doffing phase but implies that it must be fixed with tight sleeves, straps, waterproof transparent film dressing, or with tight knee-length swimsuits. During swimming a small water film between the skin and the conductive part of the silicone electrode is present. Hence, no additional hydrogel was added. Straps and knee-length swimsuits have been used in this study for the leg electrodes. The electrodes for tSCS have been fixated by waterproof transparent film dressing.

### Subjects, training protocol and outcome measures

This feasibility study was carried out at the Treatment Centre for Spinal Cord Injuries in Berlin^1^. The aim of the study was to investigate the effects of stimulation-supported swimming in two SCI patients with complete paralysis of the lower extremities after spinal trauma with a lesion above Th10. Participants have to be proficient front crawl swimmers.

Both recruited subjects (A: age 40, time since injury 10 years, B: age 58, time since injury 36 years) are ASIA impairment scale A with lesion level Th5/6 and gave written informed consent. They both complain of a moderate clonus of the lower extremities and the abdomen during position changes, and Subject A experiences leg extensor spasms from time to time. Subject B suffers from a hip joint contracture.

After the recruitment and initial assessment, the subjects were asked to carry out a four-week FES cycling training at home. During this land training, they trained at least three times a week for 30 min with a standard FES cycling ergometer (RehaMove, Hasomed GmbH, Germany). This preliminary FES cycling training was needed to build up a defined baseline strength and endurance for the swimming phase. During the swimming phase, FES cycling activity was reduced to two times a week.

The entire swim training lasted for 10 weeks. Subjects were asked to attend the weakly swim training session that lasted between 30 to 45 min (excluding donning and doffing). As a safety measure, the swim sessions were always accompanied by a trained pool guard. Furthermore, all recruited subjects are able to swim without stimulation. The training was done at a 16 m pool. Subject A used a snorkel during front crawl swimming.

Prior to the first use of tSCS during swimming, the electrode position at the spinal cord and the stimulation intensity for spasticity treatment were identified according to [8] and documented. The found constant stimulation intensity was applied in all training sessions when tSCS was on.

The stimulation amplitudes for both quadriceps were identical and have been chosen to cause an almost full knee extension while the subjects rested at the edge of the swimming pool with an upright upper body. Before each lap, the leg movement was reevaluated and the stimulation amplitude increased, if necessary, to compensate for muscle fatigue. A break of at least one minute was kept between the laps.

At the beginning of each swim training session, lap times were measured. Therefore, the subjects were instructed to swim each 16 m lap as fast as possible. When comparative measurements were taken, first the times for swimming without support were taken, then with FES support and finally the times for FES plus tSCS support. We used this order so that the results for trials with increasing amount of support are more affected by muscular fatigue then the trials with less or no support. After this initial assessment, training with the preferred support (FES or FES plus tSCS) took place for the rest of the session at self-selected swimming speed. If FES plus tSCS has been selected as preferred support, then tSCS was always active also in the breaks between the laps, while FES was switched off during these breaks.

There are three main questions that shall be answered in this pilot study:

- Does the swimming speed, assessed by lap times, increase compared to non-assisted swimming?
- Does the general well-being of the subject improve during the trial?
- How is the acceptance of the technology by the user?

The subjects were asked to rate the therapy on the basis of predefined statements using a five-grade scale between full agreement and no agreement. Using the result of the questionnaire the last two questions can be answered.

#### IMU-based motion analysis during swimming

##### Post-training assessment

Nine months after completion of the entire swim training phase, after we had acquired a suitable measurement system, we performed an additional swimming session with each of the two subjects to monitor the effects of the different stimulation programs on the leg and trunk motion. Both subjects were instructed to repetitively swim laps with no support, tSCS support, FES support, and FES plus tSCS support as fast as possible.

##### Sensor setup

**Fig. 4:**
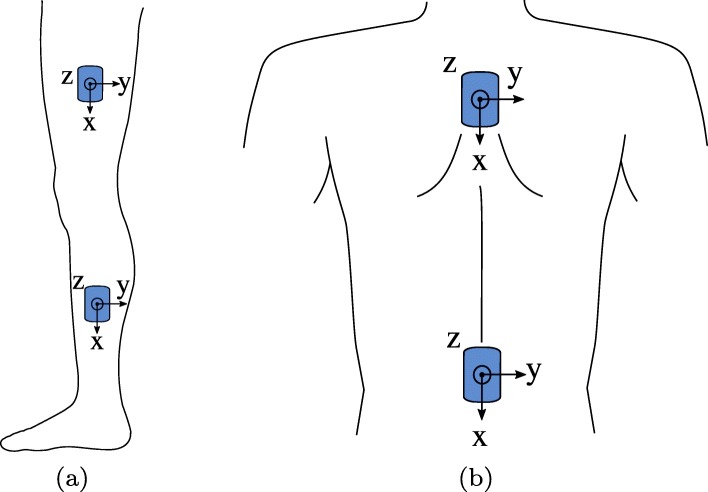
**a** IMU alignment and location on the left leg. The local x-axes are aligned with the longitudinal body axis. The z-axis points laterally to the left. **b** IMU alignment and location on the upper and lower back. The local x-axis is aligned with the longitudinal body axis, while the y-axis points to the right

A wearable sensor setup was used. The employed system WaveTrack (Cometa srl, Italy) is a wireless and waterproof inertial sensor system consisting of several time-synchronized inertial measurement units (IMUs). These inertial sensors provide three-dimensional measurements of the acceleration, angular velocity, and magnetic field vector at a frequency of 286 Hz. The sensor data were used to determine the joint angles of both knees and both hips as well as the roll orientation angles of the trunk on the cervical and lumbar level. To this end, four IMUs were bilaterally attached to the exterior thigh and shank, and two IMUs were located on the upper and lower back, as shown in Fig. 4a and b. Note that only the left leg is depicted. For both IMUs on the right leg, the local x-axis points longitudinally toward the feet, but the z-axis points laterally to the right, which implies that the y-axis points anteriorly.

As all of the sensors are located underwater during the whole measurement, wireless data transfer (streaming) is not an option. Therefore, an offline data recording is carried out. The data acquisition and time synchronization of the sensors is initiated by means of remote control. The recording begins before the subject enters the pool. After leaving the pool the recording is stopped and the data are transferred from the sensors to a PC. The software EMGandMotionTools (Cometa srl, Italy) was used for data transfer and sensor settings. Admittedly, due to the loss of communication between the sensors when located underwater, a synchronization drift is educed. However, since this drift does not exceed a few milliseconds per hour and all acquisitions last between approximately 30 to 45 min, the effect on the data is considered irrelevant.

All sensors were attached to the skin by means of double-sided adhesive tape for rough fixation. Subsequently, a transparent 3M Tegaderm film was used in order to prevent movement and loosening of the sensors during the swimming process.

##### Joint and roll angle estimation

For each body segment, the IMU readings are used to estimate the segment orientation with respect to an inertial frame of reference. To avoid the assumption of a homogeneous magnetic field inside the building and especially inside the water, we refrain from using the magnetic field vector measurements and fuse only the measured accelerations and angular rates by using a modular quaternion-based sensor fusion algorithm [32]. It must be noted that orientations obtained by such a 6-axis sensor fusion cannot be used for joint angle calculation directly since they exhibit an arbitrary heading offset and drift slowly around the vertical axis. With accurate bias estimation, that drift can be as slow as one degree in ten seconds, but it will not be reduced to perfect zero.

To overcome this drawback of the magnetometer-free approach, we exploit approximate kinematic constraints of the hip and knee joints. During the considered flutter kick motion of the legs, the hip and knee move approximately like hinge joints – flexion/extension is the dominant motion, while adduction/abduction and internal rotation occur only to a limited degree. We exploit these approximate kinematic constraints by using a recently developed relative-heading tracking algorithm [33]. That algorithm takes the orientation quaternions of both segments adjacent to the joint and corrects the heading of the distal segment’s orientation such that the joint constraint is fulfilled in a weighted least-squares sense. We apply this method repeatedly, starting from the lower-back segment and moving distally towards the shanks.

Consequently, we obtain seven quaternions that describe the body segment orientations with respect to a *common* inertial frame of reference. We can thus calculate joint angles from these quaternions. The relative joint orientations are found by multiplying the conjugate of the proximal orientation with the distal orientation. The joint angles are then calculated by intrinsic Euler angle decomposition of this relative orientation quaternion. Note that both the hip and knee extension angles are defined such that they are 180 degrees for a perfectly straight leg.

**Fig. 5:**
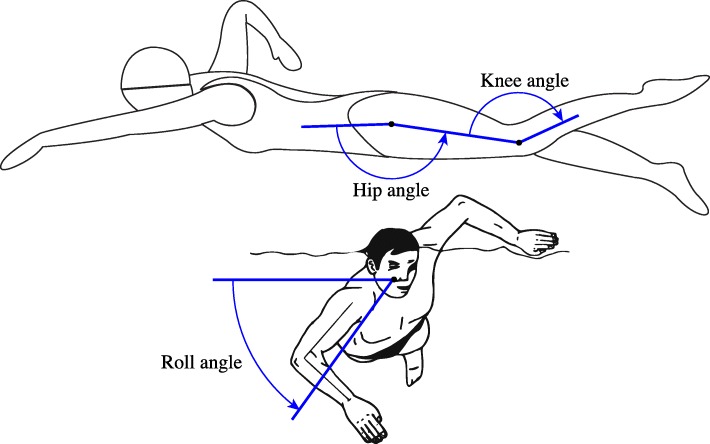
Definition of the knee and hip extension angle as well as the trunk roll angle

Finally, the roll angle of the upper and lower back is determined from the corresponding orientation quaternion. This is achieved by transforming the local left-to-right axis, i.e. the y-axis of the IMU, into the inertial frame of reference and then determining the angle between that axis and the horizontal plane, as illustrated in Fig. 5. Note that this angle is defined positive when the right side of the trunk is lower than the left side.

A segmentation of the recorded data is performed based on the norm of the 3D acceleration vector by detecting rest and motion phases. Only the first lap of each support modality is exported and investigated. From the extracted lap data, a time course over 7 strokes in the middle of the lap has been selected to analyze the joint and roll angles by using boxplots. Consequently, the start and stop phases of each lap are excluded from data analysis.

## Results

Both subjects completed the 10 weeks of training, but both subjects did not take part in all possible swim sessions due to personal reasons. Once, the pool was also not available. In total, the subjects A and B completed 6 and 7 sessions, respectively. Within each swim training, about 15 laps have been finished by each subject. After the first use of FES plus tSCS, both subjects chose this as their preferred support. Hence the major part of the training was performed with this support.

The stimulation intensities for the quadriceps for Subject A were set to initially 30% (30 mA, 150 *μs*) and then increased to up to 50% (50 mA, 250 *μs*) to compensate fatigue. Subject B started initially with 40% (40 mA, 200 *μs*) intensity, which was increased up to 60% (60 mA, 300 *μs*) depending on the fatigue state. The optimal intensity for tSCS was determined as 40 mA for both subjects. The used on/off rates for the quadriceps were 1 Hz and 2 Hz for the subjects A and B, respectively, with both subjects being asked to choose the rate at which they felt more comfortable in the first session.

**Fig. 6:**
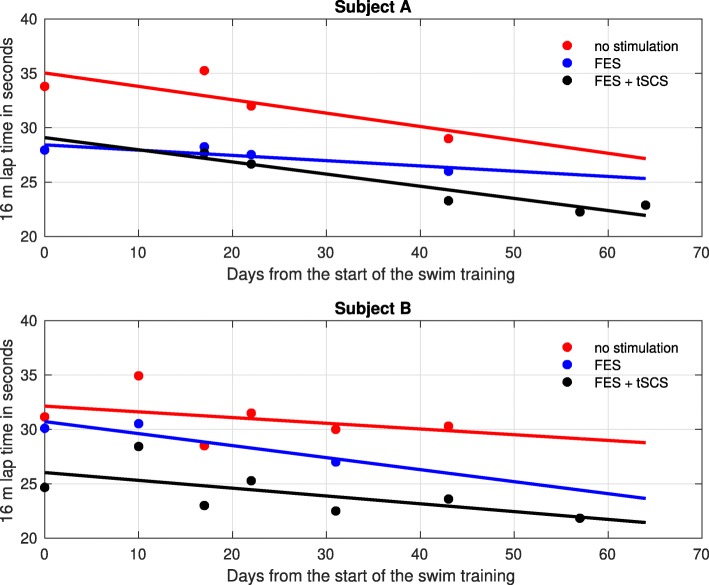
Lap times of the two subjects over the swim training phase. The lap times in a single training session have been averaged for each support modality

In Fig. 6, the mean values of the measured lap times in each training session are shown together with the calculated trend lines for all assessed support modalities (no stimulation, FES, FES plus tSCS). One to three laps have been measured in each session with the stopwatch for each investigated type of swimming support at the beginning. Tables 1 and 2 also reveal the lap times and the calculated reductions of the lap times with respect to swimming with no support when adding FES or FES plus tSCS.

**Table 1:** Lap times during the swim training phase for Subject A. The reduction in lap time with respect to the lap time without support is reported

	Average 16 m lap time [s]			Reduction [%]	
Day	No support	FES	tSCS & FES	FES	tSCS & FES
0	33.8	28.0	–	17.3	–
17	35.3	28.3	27.7	19.9	21.5
22	32.0	27.5	26.7	14.0	16.7
43	29.0	26.0	23.3	10.3	19.7
57	–	–	22.3	–	–
64	–	–	22.9	–	–
Average	32.5	27.4	24.6	15.4 ±3.6%	19.3 ±2.0%

**Table 2:** Lap times during the swim training phase for Subject B

	Average 16 m lap time [s]			Reduction [%]	
Day	No support	FES	tSCS & FES	FES	tSCS & FES
0	31.2	30.1	24.7	3.4	20.8
10	34.9	30.5	28.4	12.6	18.6
17	28.5	–	23.0	–	19.3
22	31.5	–	25.3	–	19.7
31	30.0	27.0	22.5	10.0	25.0
43	30.3	–	23.6	–	22.1
57	–	–	21.8	–	–
Average	31.0	29.2	24.2	8.7 ±4.8%	20.9 ±2.3%

The reduction in lap time with respect to the lab time without support is reported

Swimming with FES support reduced lap times by 15.4% and 8.7% on average for Subject A and Subject B, respectively. Adding further tSCS support yielded even greater mean decreases of 19.3% and 20.9% for Subjects A and B, respectively.

Additionally, both subjects individually reported that swimming with tSCS plus FES for 30–45 min completely eliminated the spasticity in the lower extremities for up to 4 h after the swim training – during this time period, it was impossible to trigger extensor spasms or a clonus by inducing postural changes. No spasticity reduction was observed after the first training session of Subject A, in which only FES support was used.

Both subjects fully agreed that FES swimming is more comfortable and enjoyable than swimming without support and that they would like to use such a device for recreational training and rehabilitation in future. They also fully agreed that swimming with stimulation is more efficient than swimming without support. Subject B states that the additional spinal cord stimulation has a positive effect on the streamlined position in the water and thus the propulsion through arms and stimulated legs is even more efficient.

The documented donning and doffing time with adhesive electrodes were approximately 10 minutes, where waterproofing the cable-to-electrode connection with 3M tegaderm film as well as careful detachment of the electrodes and the extra film required large portions of that time. The donning time was reduced to approximately 5 minutes when using the aforementioned silicone electrodes with inherent waterproof cable connection.

**Fig. 7:**
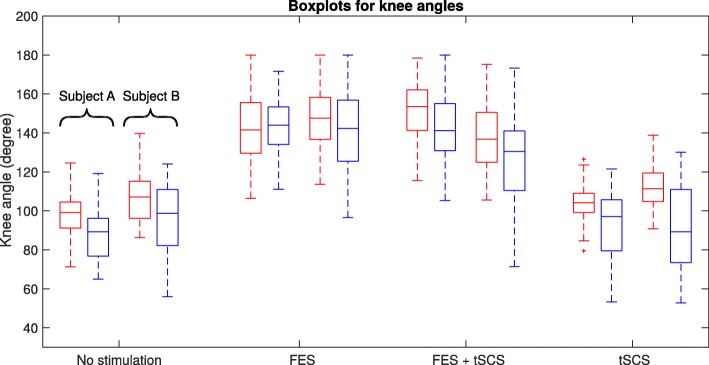
Knee joint angles during swimming without and with different support modalities (left leg – red line, right leg – blue line)

**Fig. 8:**
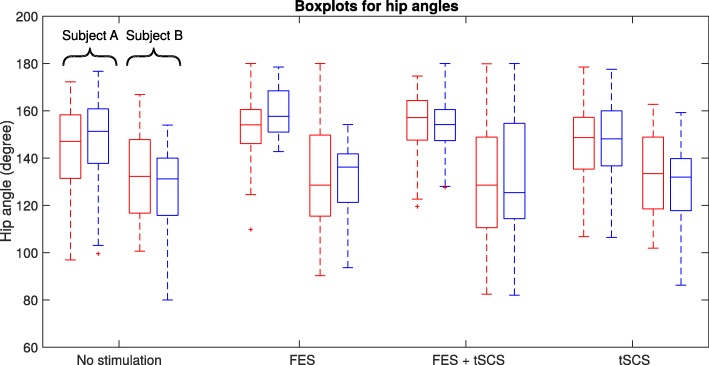
Hip joint angles during swimming without and with different support modalities (Left leg – red line, Right leg – blue line)

**Fig. 9:**
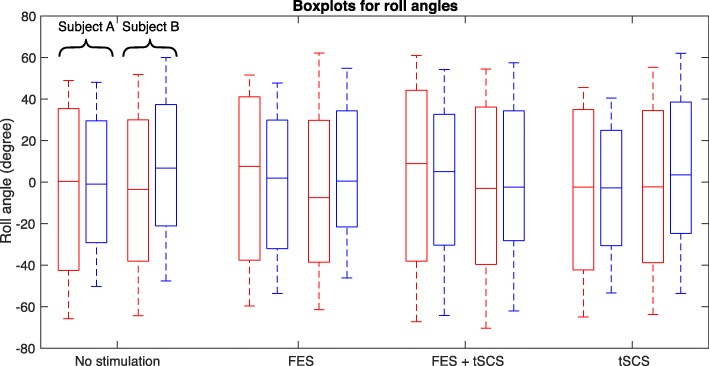
Roll angle of the lower and upper back during swimming without and with different support modalities (upper back – red line, lower back – blue line)

For the post-training assessment, in Figs. 7, 8 and 9, the distribution of the angles for each leg and the back (left – red, right – blue, upper – red, lower – blue) are summarized in boxplots for both subjects. On each box, the central mark indicates the median, and the bottom and top edges of the box indicate the 25th and 75th percentiles, respectively. The whiskers extend to the most extreme data points not considered as outliers, and the plus markers indicate the most extreme outliers.

For the knee joint angles, an increase in extension of up to 60 degrees for FES and the combination of FES and tSCS can be observed compared to swimming without stimulation. But for isolated tSCS, the angular range is not influenced. Regarding the hip joint angles, there is a minor increase of the median hip angle for Subject A and a reduced variance during FES and the combination of FES and tSCS. For Subject B no difference for the hip angle can be observed. This is in accordance with the fact that Subject B shows a manifested contracture of the hip joint.

Regarding the roll angles, no difference between the support modalities and swimming without stimulation could be measured.

The measured mean lap times during the post-training assessment with silicone electrodes are summarized in Table 3. Subject A performed every swim support type twice. Subject B fatigued early and conducted every type of swimming only once. Both subjects continued swimming with tSCS plus FES after the assessment for another 30 min at moderate speed with breaks and experienced a spastic reduction that again lasted for several hours after the swimming.

**Table 3:** Averaged lap times of the post-training assessment

	16 m lap time in seconds				
Subject	No support	tSCS	FES	tSCS & FES	Number of repetitions
A	28	30	24	22	2
B	26	34	30	31	1

## Discussion

For both subjects, a slight training effect over the training period can be recognized for swimming with and without stimulation. This was expected as both subjects are no regular swimmers. Furthermore, the results indicate that FES with and without additional tSCS can enhance the swimming speed in paraplegics during front crawl swimming. So far, the measured lap times suggest for both subjects that the greatest improvement of the swimming speed can be achieved when FES and tSCS are combined. The reasons for this are not fully understood at the moment. The IMU-based motion analysis was a first attempt to investigate this phenomenon. However, lap times for the swimming with FES and with FES plus tSCS did not differ so much in the post-training assessment, as shown in Table 3. This might explain why no differences could be observed in the joint and roll angles of the post-training assessment when adding tSCS to swimming without or with FES. The additional post training tests, therefore, provided no explanation for the tSCS effect.

The large time interval between the swim training and the later assessment with IMUs was certainly too long, but a suitable measurement system was not available before. Subject A, who conducts regular exercises, showed a similar performance as at the end of the swim training, again with minor benefits from combining tSCS and FES. Subject B, who previously gained mostly from additional tSCS, did not train at all after the end of the swim training, and was, as a result of it, quickly exhausted already during the first laps. His physical condition at the post-training assessment was certainly not comparable to the one nine months before. The progressing fatigue during the four laps with time taking has probably affected the results. It seems that the improvements made by FES and FES plus tSCS have just compensated for the performance losses caused by fatigue.

A possible explanation for the earlier observed positive effects of tSCS during swimming training could be that trunk stability continuously improved by tSCS, and as a result, reduced the swimmer’s necessary effort to avoid trunk rolling. But also placebo effects can not be ruled out at present, as no sham tSCS stimulation was applied. Theoretically, subjects may have unconsciously swum much more with their non-paralyzed arms if they knew and felt that tSCS was active.

The use of different electrodes during the training period and the post-training assessment might be interpreted as another weakness of the study design. However, the observed lap times in Subject A and spasticity reduction for both subjects with the silicone electrodes indicate, that this simply applicable type of electrodes is suitable for stimulation in water and yields comparable effects as with adhesive electrodes with oversized waterproof backing.

No synchronization between the arm and leg movement has been realized in this study. Such a synchronization might help to prevent undesired rolling movements of the swimmer and could have additional effects on the swimming speed. In the case of crawl stroke, the knee extension should be synchronized with the contralateral arm movement to increase swimming speed and effectiveness. The first approaches for this synchronization problem of arm and leg movement have been presented by us recently. An electrotactile biofeedback that informs the swimmer about the leg movement was proposed first [25]. Unfortunately, swimmers could not concentrate on this feedback channel during swimming and had problems to adapt the pace of their arms to the technically dictated pace of the legs. More promising seems the approach to indirectly detect the arm movement via measurements of the trunk roll angle and to trigger the stimulation based on this motion signal [22]. That solution should assure that the stimulation is only active during swimming and that the user is able to activate and deactivate the stimulation of the legs by trunk motions. Validation of the approach with patients is still pending. At the moment, the stimulation patterns are very crude and heuristically derived. The potential for pattern optimization and arm-leg synchronization to increase swimming speed cannot be assessed currently.

Major imitations of this feasibility study were the small number of subjects and the not so rigorous training and assessment protocol. The individual contribution of swimming and FES cycling to the observed training effect can also not be quantified. Another weakness of the current study is the way how the swimming speed is assessed. The usage of the lap time is error-prone especially for short tracks, as the start technique and the occasional presence of extensor spasms at the beginning of a track have a strong influence. A larger pool would be advisable or better methods, for example IMU-based velocity estimation, to determine the instantaneous speed during swimming. Additional IMUs at the arms should be used to rate the involvement of the arms for propulsion and stabilization of the roll angle. Another important factor is the metabolic efficiency, which has not been studied at all in the present work.

## Conclusions

A new hybrid exercise modality for SCI patients has been proposed that involves the voluntarily moved arms and the artificially stimulated legs and trunk. The swimming exercise can be performed independently by the patient without any additional assistance. The results in two complete ASIA scale A subjects showed that the swimming speed during front crawl could be increased using electrical stimulation either with FES or with the combination of FES and tSCS. The latter yielded better results in both subjects.

The prolonged use of FES plus tSCS caused a long lasting reduction of spasticity in both subjects. To better quantify and document this effect in the future, measurement systems for automatic spasticity assessment are required (e.g. to count the occurrence frequency of a clonus).

Future work should aim at optimizing the stimulation pattern based on the IMU-based leg motion measurements – or eventually at building closed-loop systems that adjust the stimulation intensities in real time in order to achieve the desired movement also in the presence of disturbances and muscular fatigue. Solutions from FES-cycling and -rowing might be adapted for this problem, and segmentation of the swimming phases might prove useful.

To further improve the usability of the swimming device, we plan to incorporate silicone electrodes with all cables inside a neoprene sleeve. This could further reduce the donning and doffing time and increase safety since cables would be less prone to entangle or to slip off the electrodes.

The research is still in an early phase, and further tests with more subjects are needed to quantify, analyze and improve the training effects for the SCI patients, looking also at the support of back stroke swimming by stimulation for paraplegics and tetraplegics who are not able to perform front crawl swimming.

As pointed out throughout the introduction, hydrotherapy is a promising therapy for neurological rehabilitation in incomplete SCI, stroke, or multiples sclerosis patients. Therefore, further studies shall incorporate other neurological disorders and investigate potential benefits of FES and tSCS for gait and balance therapy in water. The use of the presented technology for paraplegic scuba diving is another promising recreational application.

## Supplementary information

**Additional file 2** Subject B.

## Data Availability

The data set used and/or analyzed during the current study is available from the corresponding author upon reasonable request.

## Abbreviations

FES: Functional electrical stimulation
IMU: Inertial measurement unit
tSCS: Transcutaneous spinal cord stimulation

## Footnotes

1 Ethical approval of Berlin Chamber of Physicians Eth-28/17
